# A scoring strategy combining statistics and functional genomics supports a possible role for common polygenic variation in autism

**DOI:** 10.3389/fgene.2014.00033

**Published:** 2014-02-18

**Authors:** Jérôme Carayol, Gerard D. Schellenberg, Beth Dombroski, Claire Amiet, Bérengère Génin, Karine Fontaine, Francis Rousseau, Céline Vazart, David Cohen, Thomas W. Frazier, Antonio Y. Hardan, Geraldine Dawson, Thomas Rio Frio

**Affiliations:** ^1^IntegraGenEvry, France; ^2^Department of Pathology and Laboratory Medicine, University of PennsylvaniaPhiladelphia, PA, USA; ^3^Groupe Hospitalier Pitié-Salpêtrière, Department of Child and Adolescent Psychiatry, AP-HP, Université Pierre et Marie CurieParis, France; ^4^Center for Pediatric Behavioral Health and Center for Autism, Cleveland ClinicCleveland, OH, USA; ^5^Department of Psychiatry and Behavioral Sciences, Stanford UniversityStanford, CA, USA; ^6^Department of Psychiatry and Behavioral Sciences, Duke University Medical CenterDurham, NC, USA

**Keywords:** autism, genetic variance, polygenic model, common variants, genetic score, functional genomics

## Abstract

Autism spectrum disorders (ASD) are highly heritable complex neurodevelopmental disorders with a 4:1 male: female ratio. Common genetic variation could explain 40–60% of the variance in liability to autism. Because of their small effect, genome-wide association studies (GWASs) have only identified a small number of individual single-nucleotide polymorphisms (SNPs). To increase the power of GWASs in complex disorders, methods like convergent functional genomics (CFG) have emerged to extract true association signals from noise and to identify and prioritize genes from SNPs using a scoring strategy combining statistics and functional genomics. We adapted and applied this approach to analyze data from a GWAS performed on families with multiple children affected with autism from Autism Speaks Autism Genetic Resource Exchange (AGRE). We identified a set of 133 candidate markers that were localized in or close to genes with functional relevance in ASD from a discovery population (545 multiplex families); a gender specific genetic score (GS) based on these common variants explained 1% (*P* = 0.01 in males) and 5% (*P* = 8.7 × 10^−7^ in females) of genetic variance in an independent sample of multiplex families. Overall, our work demonstrates that prioritization of GWAS data based on functional genomics identified common variants associated with autism and provided additional support for a common polygenic background in autism.

## Introduction

Autism Spectrum Disorders (ASDs) are characterized by impairments in social interaction and communication, restricted interests, and repetitive behaviors with a 4:1 male to female ratio (Johnson and Myers, 2007). A recent systematic review estimated a median ASD prevalence of 62/10,000 globally and 65.5/10,000 in the US and Canada (Elsabbagh et al., 2012). Based on a standardized assessment of description of behaviors from administrative or health records, not on standardized diagnostic interview in the general population, the Centers for Disease Control estimated in 2012 that up to 1 in 88 children have an ASD (Wingate et al., 2012).

The recurrence risk in siblings of children with ASD is estimated to be 18.7% (Ozonoff et al., 2011), which is 16 times higher than in the general population. Earlier twin studies have reported pairwise concordance rates for monozygotic (MZ) twins in the range of 36–96% while in dizygotic (DZ) twins this rate was lower than 30% resulting in heritability estimates higher than 90% (Folstein and Rutter, 1977; Steffenburg et al., 1989; Bailey et al., 1995; Farley et al., 2009). The latest twin study to date confirms this high heritability with 95.2% concordance rate in MZ twins and 4.3% in DZ twins (Nordenbaek et al., 2013). To contrast these results, one recent study provided a lower estimate of heritability (37%) but with wide confidence intervals (CI) (8–84%) (Hallmayer et al., 2011). As many as 15% of cases may be attributable to rare genetic factors like *de novo* mutations, rare copy number variations (CNVs) or chromosomal abnormalities but several common variants including CNVs and single nucleotide polymorphisms (SNPs) have also been strongly linked to autism (Cook and Scherer, 2008; Freitag et al., 2010; Devlin and Scherer, 2012). The contribution of rare and common variants to autism and their possible interactions remain to be determined, but available evidence suggests that common variants, despite each not being causal, increase the susceptibility to the disorder (Abrahams and Geschwind, 2008; Klei et al., 2012; Stein et al., 2013). There is an increasing interest in making predictions of complex traits phenotypes from genetic information (Manolio, 2013). With the availability of genome-wide data, many common variants have been identified in complex diseases like cancers. Based on these results, common polygenic models are tested for their discriminatory power and potential application in screening programs (Pharoah et al., 2008; So et al., 2011). To date, much of the ASD risk information is complex and ethical concerns have been recently discussed (Rossi et al., 2013). However, prediction may positively impact patient outcome by contributing to an earlier diagnosis of autism, thereby providing earlier access to services. Based on a small number of common variants, polygenic models has recently been proposed to estimate autism risk (Carayol et al., 2011). Assuming that thousands of common variants may explain more than 40% of autism genetic variation, one could expect a high predictive power of a polygenic model synthesizing their information (Klei et al., 2012).

Several genome-wide association studies (GWASs) have been performed to decipher the genetic etiology of autism that is attributable to common variants (i.e., SNPs) with only a few variants having shown significant associations and replicated in an independent population or in endophenotypes (Wang et al., 2009; Weiss et al., 2009; Anney et al., 2010; Connolly et al., 2012). Considering their small effect, these SNPs represent only a small proportion of the large number of common variants required to reach the reported high heritability estimates (Klei et al., 2012). By virtue of their joint effect, SNPs that do not reach the stringent genome-wide significance level in GWASs may be biologically important nonetheless (Meuwissen et al., 2001; Wray et al., 2007), and their information, if combined in genetic risk models, could be used to estimate the genetic susceptibility to complex diseases (Purcell et al., 2009; Bush et al., 2010; Levinson et al., 2012; Stahl et al., 2012). To overcome the lack of power inherent in GWASs and to decipher the common polygenic background of complex disease, novel methods (e.g., statistical noise reduction or gene ontology enrichment) have emerged that make it possible to prioritize results that did not reach statistical significance in autism studies (Anney et al., 2011; Hussman et al., 2011). In autism, a pathway-based approach was proposed to prioritize SNPs from GWAS data and combine them in a genetic classifier (Skafidas et al., 2012). Convergent functional genomic (CFG) analysis is an alternative gene-level analysis method that is used to prioritize common variants that did not reach the significance level in GWAS, based on the function of the genes in which they stand and the potential impact on the disease, through the integration of multiple lines of biological and genetic evidence (Le-Niculescu et al., 2009; Patel et al., 2010; Ayalew et al., 2012). This strategy was demonstrated to be successful in studies of bipolar disorder and schizophrenia in which SNPs, despite displaying weak statistical signals, were selected on the basis of gene-based analyses. Combined in a genetic risk prediction score, these prioritized common variants were demonstrated to discriminate affected subjects from healthy controls (Patel et al., 2010; Ayalew et al., 2012), while polygenic models based on SNPs selected according to simple *P*-value criteria from GWAS provide evidence about the role of common variants in the disorders (up to 3% of genetic variance explained) but have little value for risk prediction (Purcell et al., 2009).

Using a discovery sample of multiplex families from the Autism Genetic Resource Exchange (AGRE) (Lajonchere, 2010), we aimed to identify SNPs that were overtransmitted from parents to affected or unaffected children and which also discriminate children with autism from their unaffected siblings (Sacco et al., 2007). For this purpose, a GWAS that combined association information from parent-offspring transmission and a comparison of affected-unaffected siblings was performed. Because there is significant sexual dimorphism of the genetic risk for autism, ignoring sex effects lessens the statistical power and could lead to the failure to identify a significant proportion of the genes that contribute to risk (Ober et al., 2008; Lu and Cantor, 2012). Through adding evidence of sex-specific risk alleles, genetic models, and variable penetrance of alleles in ASD, we performed the GWAS with and without sex stratification (Stone et al., 2004; Schellenberg et al., 2006; Weiss et al., 2006; Sato et al., 2012). A scoring approach similar to CFG was then applied to extract genome-wide association signals from “noise.” The scoring algorithm of the prioritization method integrates statistical characteristics from the GWAS with functional genomic evidence.

Using an independent validation population of multiplex families, SNPs that were detected within the discovery population were selected and combined in a sex specific genetic score (GS). The GS was tested for association in this validation sample and the proportion of genetic variance explained evaluated.

## Materials and methods

### Participants and genotyping

**Figure 1:**
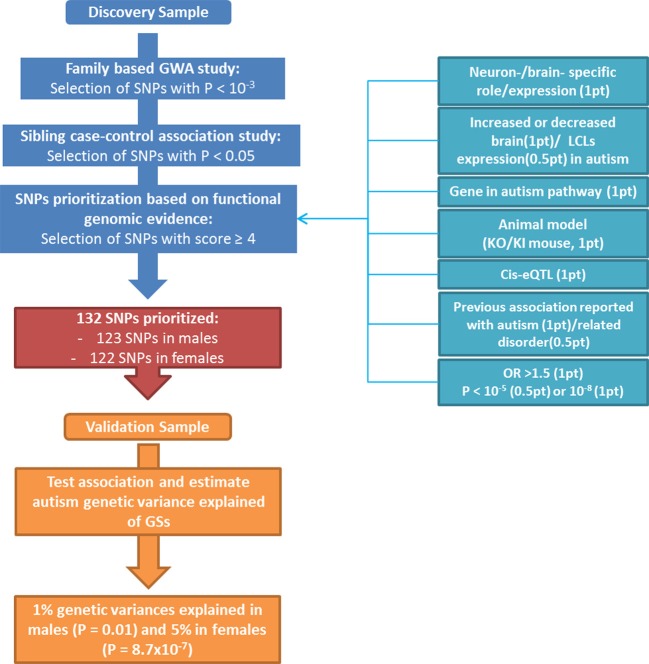
**Flow chart displaying the different steps for SNPs prioritization with details of the scoring strategy**.

Two independent sets of DNA samples from autism multiplex families were used in this study (Figure 1). The discovery population consisted of 545 multiplex families from AGRE repository and included 964 affected siblings (773 males, 191 females; 4.1:1 male-to-female sex ratio) and 317 unaffected siblings (144 males, 173 females). The validation population consisted of 288 multiplex families from a collection at the University of Pennsylvania (originally collected at the University of Washington) that was enriched with a complementary set of 339 families from AGRE (independent from the discovery sample). It was composed of 1,000 affected siblings (812 males, 188 females; 4.3:1 male-to-female sex ratio) and 288 unaffected siblings (141 males, 147 females). The diagnosis of autism was made using the standard Autism Diagnostic Interview-Revised (ADI-R) algorithm (Lord et al., 1994). Only individuals with a “strict” definition of autism (93 and 86% of children with ASD in the discovery and validation populations, respectively), including individuals who exceeded the ADI-R cut-off for autism in all domains as defined in the AGRE repository (http://www.agre.org/), were selected to improve the power of the GWAS by homogenizing the phenotype (Shao et al., 2002; McCarthy et al., 2008). Members of the AGRE families were genotyped using the Infinium II HumanHap550 BeadChip at the Center for Applied Genomics at The Children's Hospital of Philadelphia (CHOP), as previously described (Wang et al., 2009). SNPs that failed the Hardy Weinberg Equilibrium Test (*P* < 10^−3^) or that had a call rate less than 90% or a minor allele frequency less than 5% were removed. Mendelian transmission of alleles was checked for every SNP; genotypes that were inconsistent with Mendelian inheritance in one or several families were considered as unknown in all members of the families showing the error. SNPs identified in the discovery population were genotyped in the University of Pennsylvania collection, as previously described (Carayol et al., 2011).

### Association studies

In the discovery population of 545 multiplex families from AGRE, family-based association tests were performed using the Family Based Association Test (FBAT) software (Laird et al., 2000) under additive, recessive, and dominant inheritance models to maximize power (Burton et al., 2007; Kang et al., 2010). FBAT tests for an excess of an individual SNP allele from parents to affected siblings. The interaction of sex with genotype may manifest in a genotypic effect that is apparent only in affected females, only in affected males, or in both sexes but with different magnitude or direction of effect (Strohmaier et al., 2013). Furthermore, the difference in ASD prevalence may result in male- and female- specific genes, or, more likely, from the differential penetrance of some risk alleles on the basis of the sex of the affected individual, leading to a lower power to detect existing weak effects in a particular gender stratum, compared with a higher effect in the other stratum (Hirschhorn et al., 2002; Schellenberg et al., 2006; Lu and Cantor, 2012). Consequently, affected male and female siblings were analyzed together and then independently. Because of the potential for protective alleles in autism pathogenesis, the transmission of markers from parents to unaffected siblings was also assessed (Sacco et al., 2007). SNPs associated with a *P*-value < 10^−3^ were evaluated for their ability to discriminate individuals with autism from their unaffected siblings through a sibling case-control association analysis under the same gender specificity. Odds ratios (ORs) were estimated using a generalized estimating equation (GEE) model with an independence correlation matrix to account for the non-independence of individuals from the same family (Zeger and Liang, 1986; Hancock et al., 2007). Gender was introduced as an adjustment covariate when it was not used as a variable of stratification. SNPs associated at the nominal level were selected for further analysis.

### SNP-gene pair prioritization

Many association tests were performed based on different genetic models and gender stratification to extract a maximum of the association signals but at the cost of an inflated number of false positive results. One way to minimize these false positive results consists of correcting *P*-values for multiple testing. At the genome-wide level, a 5 × 10^−8^ *P*-value threshold is generally used to define an association between a SNP and the disease as significant. Unfortunately, such criterion is too stringent in GWA study of complex disease to identify low risk common variants in samples of moderate size like in autism. To extract association signals from GWAS and minimize false-positive results, we developed a gene-based scoring method based on CFG, in which points were allocated to SNPs as indicated in Table 1. “Related gene” refers to the nearest gene to the SNP (± 5 kb upstream and downstream) or, when no gene matched this criterion, both the closest downstream and upstream genes within 50 kb of the SNP. For scoring, information was integrated from previous reports of SNP-related genes that may be linked to autism and to other neurodevelopmental disorders demonstrated to share a common genetic background [schizophrenia, bipolar disorder, and mental retardation (Ben-Shachar et al., 2009; Carroll and Owen, 2009; Berkel et al., 2010; Crespi et al., 2010)] as well as from genomic characteristics of the scored SNP (SNP location and eQTL property). Human data from autism brain gene expression and lymphoblastoid cell line expression studies were also considered in order to determine the specific role or expression of the SNP-associated gene in the central nervous system (CNS) (Purcell et al., 2001; Garbett et al., 2008). Involvement of the SNP-related gene in a pathway implicated in ASD was jointly integrated with data about impairment of the CNS or function in knockout models such as mouse. The maximum score for a gene was 9 (Table 1). SNPs were selected for further analyses if they displayed a score greater than or equal to 4. This cut-off was chosen based on observation of all SNPs scored and the distribution of points in the different categories. Only SNPs with a score above this cut-off displayed points allocated to statistical (“Statistical Parameters” in Table 1) and functional genomic evidences.

**Table 1:** **Prioritization and scoring algorithm rules (details are given in Supplementary Table 2) for SNPs selected with *P* < 0.001 in the family-based GWA and *P* < 0.05 in the siblings case-control studies performed in the discovery sample**.

**General principle**	**Points**	**Example**
**Statistical parameters (max 2 points)**		
*P*-value of the association of the SNP with autism in GWAS is <10^−8^ vs. between 10^−8^ and 10^−5^.	1 vs. 0.5	None^*^ vs. rs7974275 (GRIN2B)
Odds ratio associated with the risk-associated allele of the SNP is ≥1.5 in the sibling case-control study.	1	rs4251859 (PLAUR)
**Genomic characteristics (max 2 points)**		
The SNP is located within the gene (including 5 kb upstream and downstream regions).	1	rs2770298 (HTR2A)
The SNP acts as an eQTL of the gene as determined by two eQTL databases, “Genevar” (Yang et al., 2010) and “eQTL resource @ Pritchard's lab” (eqtl.uchicago.edu) (Veyrieras et al., 2008; Degner et al., 2009; Pickrell et al., 2010).	1	rs2297389 (GABRR1)
**Previous reporting (max 1 point)**		
The gene has been associated through genome-wide or gene candidate association studies, mutations, or structural abnormalities with autism vs. with a related neurodevelopmental genetic disorder (e.g., schizophrenia, bipolar, mental retardation).	1 vs. 0.5	rs3928471 (SLC9A9) vsrs72723811 (NRG1)
**Physiological properties (max 4 points)**		
The expression of the gene is significantly different in patients with autism compared with controls in brain (Purcell et al., 2001; Garbett et al., 2008) or in lymphoblastoid cell lines (Gregg et al., 2008; Hu et al., 2011).	1 vs. 0.5	rs3928471 (SLC9A9) vs. rs636624 (PTPRG)
The gene has a specific role or restricted expression in the CNS.	1	rs12514116 (KCNIP1)
A mouse model exhibits either impairment of CNS development or function with or without an autism-related behavior as reported in the mouse gene informatics database from the JAX laboratory (Blake et al., 2011) and literature.	1	rs314253 (DLG4)
The gene is a part of a pathway in which other genes have been strongly associated with autism (development of the CNS, neurogenesis, neuronal migration, neuron projection, synaptogenesis, synaptic transmission) or is a part of a biochemical pathway from in which other genes have been strongly associated with autism (e.g., TSC/mTOR, MET receptor tyrosine kinase pathways).	1	rs9940922 (CDH13)

### Definition of the genetic models and development of GSs

SNPs fulfilling statistical and functional genomic criteria in the discovery analysis were used to construct a GS. Sex-specific GSs of individuals were built in the discovery sample as the sum of deleterious alleles under their best fitted genetic model (Carayol et al., 2011). The “best-fitted” model was defined as the one that maximized the proportion of genetic variance in the discovery population when all SNPs were considered in a polygenic model. When only SNPs with consistent direction of allelic association were analyzed, genetic models without consistent direction of effect were excluded from the “best-fitted” model evaluation. The proportion of genetic variance explained by the GSs was estimated as in Wray et al. (2010) according to the most recent prevalence of autism in the United States, estimated to be 1 in 88 children (1 in 54 males and 1 in 252 females) and recurrence risk in siblings estimated to be 25.9 and 9.6% in male and female siblings, respectively in a large, international, multicenter, prospective study in siblings of children with ASD (Ozonoff et al., 2011; Wingate et al., 2012).

The GS model was evaluated in the validation sample testing its association to the disorder and estimating the genetic relative risk (GRR) using a GEE in males and females separately. Inflation effect on GRRs because of SNPs selection according to consistent direction of allele effect in the validation sample was evaluated based on 1,000 bootstraps resampling (Efron and Tibshirani, 1986). Mean of GRR values estimated in each bootstrap sample was then compared to the GRR observed value. Empirical 95% CIs were determined by bootstrapping 1000-times the validation sample using each family as a resampling unit.

## Results

A flow chart illustrating the identification and prioritization of SNPs, as well as the validation processes followed in this study, is shown in Figure 1. A set of 900 SNPs (Table 1 in supplementary materials) was identified from the family-based GWAS performed in the discovery population with risk allele associated at the nominal level in the sibling case-control study. This set of SNPs was then prioritized through the selected scoring strategy. One SNP, rs11123610 in the allantoicase gene, was statistically associated at the genome-wide level (*P* < 1.3 × 10^−8^ assuming a 5 × 10^−8^ genome-wide significativity threshold and a Bonferroni correction for four different GWA studies) under a recessive model with *P* = 8.8 × 10^−9^. Allantoicase participates in the uric acid degradation pathway, but its activity is absent in mammals (Vigetti et al., 2002). Based on statistical evidence, this SNP should be considered for replication; however, the corresponding gene did not provide any functional evidence of relation to autism and was thus discarded by our scoring criteria as a likely false-positive association. Among genes related to SNPs identified through the GWAS, 330 were associated with some functional evidence in human and/or animal models (at least 1 point from “Physiological properties,” Table 1), suggesting potential involvement in autism. After prioritization of SNP-gene pair scores, a subset of 133 SNPs related to 119 genes had a score greater than 4 (Supplementary Table 2). The highest score was 7 for a unique SNP-gene pair, which was observed for rs4251859 related to *PLAUR*, the gene encoding the plasminogen activator, urokinase receptor. This gene has previously been described as an autism-risk gene in a large association study and has also been linked to autism based on strong functional genomic evidence from animal models and expression studies in brains from patients with autism (Powell et al., 2003; Eagleson et al., 2005; Campbell et al., 2008; Garbett et al., 2008).

One SNP was discarded from subsequent analyses because of genotyping failure in the validation population. From the subset of 132 SNPs selected by the scoring strategy (Table 3 in Supplementary materials), 123 were associated to the disorder in males and 122 in females in the Discovery sample before selection based on consistency of direction of allele effect. They respectively explained 1 and 5% of the genetic variance in the validation sample. GSs were significantly associated with autism in males (*P* = 0.01) and in females (*P* = 8.7 × 10^−7^).

## Discussion

The scoring strategy utilized in the present study prioritized a set of 132 independent common variants with low evidence of association in a GWAS that are related to 119 genes with potential links to autism. Combined in a sex-specific GS, these common variants explained 1% of the genetic variance in males and 5% in females, producing additional support for a common polygenic background in autism and the role of common variants as a part of risk for developing autism.

The present gene-based scoring approach was inspired by CFG, which has been previously shown to identify genes and pathways implicated in bipolar disorder (Le-Niculescu et al., 2009)—some of which have been confirmed in independent studies (McGrath et al., 2009)—and schizophrenia (Ayalew et al., 2012). To determine the score of a SNP-gene pair, points were allocated to the statistical characteristics of the SNP that reflected its discriminative ability. Points were also allocated to the related gene according to its association with autism and/or its role in the CNS. For example, several SNPs received points since they were located within the locus of known autism-susceptibility genes such as *PLAUR*, *HTR2A*, *RORA*, *CADM1*, *SLC9A9*, *GRIN2A*, *GABRA4*, *GABRB1, RBFOX1*, and *PCDH10*. Although the vast majority of scored genes had no previous evidence (significant association, mutations, differential expression) of a link with autism, some had been previously linked to genetically-related neuropsychiatric disorders such as schizophrenia and bipolar disorder and may be potentially implicated in autism, as has been demonstrated for genes such as *HTR2A*, *GRIN2A*, and *RBFOX1* (Carroll and Owen, 2009). Additionally, in light of the abnormal brain development and functioning observed in autism, the vast majority of identified genes were prioritized since they have a predominant role in the CNS and its functioning (Courchesne et al., 2007; Polsek et al., 2011). Genes with a specific role in the major processes that are altered in autism (development of the CNS, neurogenesis/neuronal migration/neuron projection, and synaptogenesis/synaptic transmission) were allocated more points to reflect their greater potential for a role in autism etiology (Bauman and Kemper, 2005; Geschwind and Levitt, 2007; Bourgeron, 2009; Wegiel et al., 2010; Melom and Littleton, 2011). The precise function of each gene was determined through a careful review of the literature including the analysis of knockout mouse models, a crucial step in the determination of the gene's function during brain development.

An allele score approach based on thousands of SNPs selected using *P*-value criteria in a GWA study explained up to 0.78% of autism variance (Anney et al., 2012). The scoring strategy alone or combine with a selection of less than a hundred of SNPs according to consistency of their allelic effect also extract a non-negligible proportion of genetic variance. Although a different cohort from the Autism Genome Project was used in their study, our results provide some evidence that the scoring strategy allow to extract true association from noise and reduce the number of selected SNPs from thousands to hundreds of informative SNP.

A set of SNPs identified during a discovery association study may include false-positive results that will outweigh the effects from true variants, decrease the predictive accuracy, and subsequently decrease the explained genetic variance (Wray et al., 2010). Such a SNP set could be enriched for true positives compared with false positives by increasing discovery sample size (Evans et al., 2009). However, because of the small effect size of common variants, a sufficiently large sample size may not be practical for autism at this time because of the limited number of multiplex families with available genotypic information. The scoring strategy is a powerful alternative to reduce the “noise” variants by excluding SNPs in genes without functional evidence. Moreover, the proportion of genetic variance explained by GWAS data increased when the *P*-value association threshold for autism was increased from 10^−5^ to 0.5, similar to observations in related neurodevelopmental disorders, which suggests that thresholds used in the present GWAS could be relaxed to enrich the GS models with true variants (Purcell et al., 2009; Anney et al., 2010).

A pathway-based approach was proposed in autism to prioritize SNPs from GWAS and design a polygenic score that could predict autism diagnosis with good accuracy (Skafidas et al., 2012). This method only used pathway data while our scoring strategy combined their information with other biological and genetic evidences. A set of SNPs was identified in a first case-control sample and used to predict autism diagnosis with good accuracy in a second independent sample. Despite an interesting statistical approach to generate a common polygenic model in autism, results were biased because population structure of cases and controls was not considered (Belgard et al., 2013; Robinson et al., 2013).

Population structure may confound genetic classifiers as demonstrated in autism (Belgard et al., 2013; Robinson et al., 2013). However, use of unaffected siblings in association studies (instead of independent controls) protects against false positives resulting from population stratification (Dempfle et al., 2008). So, to maintain statistical power, affected, and unaffected individuals from the different families were analyzed as a whole without considering ethnicity, but limiting false positive results coming from population stratification using unaffected siblings as controls.

Although not in the scope of our study, it would have been of interest to better characterize the true underlying genetic model of the common variants. Reproducibility of the genetic models for each common variant was compared in the 2 populations using a bootstrap reproducibility index (RI) (Ma, 2006; Carayol et al., 2011). A subset of 57 SNPs with highly reproducible genetic models (RI > 80%) in both populations explained a large proportion of the genetic variance in the validation population (data not shown).

We acknowledge that different ways of scoring SNP-gene pairs could have given slightly different results. Nevertheless, the simple scoring system that was developed in this study provides a good prioritization of SNPs with regard to our focus of mining real statistical signals from “noise” in the GWAS. The major limitation of the proposed scoring approach is its dependence on available information about genes; however, its power will increase with the evolution of knowledge.

In conclusion, adding function evidence of genes to GWA results is a powerful alternative to allele score approach in order to identify common variants associated to autism. In a sample of moderate size, the scoring strategy like the CFG method allow to extract and prioritize biologically relevant signals when classical GWA studies based on *p*-value selection only identified a limited number of SNPs. Common variants displaying liberal association *p*-value (*p*-value > 0.05) explain a proportion of genetic variance in autism (Anney et al., 2012) like in schizophrenia and bipolar disorder (Purcell et al., 2009; Levinson et al., 2012). Apply the scoring strategy to SNPs selected with liberal *p*-value from GWAS could identify additional genes and enhance our understanding of the common genetic basis of autism.

## Author contributions

Jérôme Carayol and Thomas Rio Frio participated in the design of the study, the analysis of data, the interpretation of data, and to draft the manuscript. Beth Dombroski, Bérengère Génin, Karine Fontaine, Francis Rousseau, and Céline Vazart participated to the genotyping of the samples and the interpretation of data. Claire Amiet, David Cohen, Thomas W. Frazier, Antonio Y. Hardan, Gerard D. Schellenberg, and Geraldine Dawson participated to the interpretation of data, and to review the manuscript. All authors read and approved the final manuscript.

### Conflict of interest statement

Jérôme Carayol, Claire Amiet, Karine Fontaine, Bérengère Génin, Francis Rousseau, Céline Vazart, and Thomas Rio Frio are currently salaried employees of IntegraGen. Jérôme Carayol, Francis Rousseau, and Thomas Rio Frio have patent applications with IntegraGen. Gerard D. Schellenberg and Beth Dombroski declare that they have no competing interests; Antonio Y. Hardan, David Cohen, and Geraldine Dawson are compensated consultants for IntegraGen. Thomas W. Frazier has received a clinical research grant from IntegraGen.
